# Feasibility and safety of modified en-bloc resection in endoscopic thyroid surgery via bilateral areolar approach – long-term institutional analysis ten years after surgery

**DOI:** 10.3389/fendo.2024.1302510

**Published:** 2024-04-17

**Authors:** Qiuping Xie, Junjie Ma, Yu Du, Lianxuan Liu, Ruiying Zhu, Danni Liu, Ping Wang, Xing Yu

**Affiliations:** ^1^ Department of Thyroid Surgery, The Second Affiliated Hospital of Zhejiang University School of Medicine, Hangzhou, China; ^2^ College of Medicine, Zhejiang University, Hangzhou, China

**Keywords:** thyroid cancer, endoscopic surgery, postoperative complications, vocal cord paralysis, hypoparathyroidism

## Abstract

**Purpose:**

This study aimed to introduce a new modified en-bloc resection method and evaluate its feasibility and safety in endoscopic thyroid surgery via bilateral areolar approach (BAA).

**Methods:**

Papillary thyroid carcinoma (PTC) patients who underwent lobectomy and ipsilateral central node dissection (CND) via the BAA approach were retrospectively reviewed. Their clinical characteristics and outcomes were evaluated, including operative duration, lymph node yield (LNY), surgical complications, recurrence rate, and metastasis rate, over a ten-year follow-up period. Simultaneous lobectomy and CND were performed in the modified en-bloc group, whereas lobectomy was performed first, followed by CND in the conventional group.

**Results:**

The study included 108 patients in the modified en-bloc group and 213 in the conventional group. There were no significant differences in gender, age, tumor locations, tumor dominant nodule size, or the incidence of concomitant Hashimoto thyroiditis when comparing clinicopathologic characteristics. The comparison of operative duration (P = 0.14), blood loss (P = 0.13), postoperative hospital stay (P = 0.58), incidence of transient vocal cord paralysis (P = 0.90) and hypocalcemia (P = 0.60) did not show any differences. The mean LNY achieved in the central compartment of the modified en-bloc group (7.5 ± 4.5) was significantly higher than that in the conventional group (5.6 ± 3.6). Two patients in the modified en-bloc group and two in the conventional group experienced metastasis after surgery during the ten-year follow-up (1.8% vs. 0.9%, P = 0.60). The learning curve analysis showed a significant decrease in operative duration after the 25-35^th^ cases for modified en-bloc resection.

**Conclusions:**

The modified en-bloc resection method in endoscopic thyroid surgery via BAA is a technically feasible and safe procedure with excellent cosmetic outcomes for selective PTC patients.

## Introduction

Over the past decade, there has been a significant increase in the incidence of thyroid carcinoma (TC), particularly papillary thyroid carcinoma (PTC), globally (1). A statistical assessment of net drift, which measures the overall annual percentage change over time adjusted for age groups, revealed an incidence of 5.01% for men and 1.48% for women (2). This incidence is projected to increase by 32.4% in men and 13.1% in women from 2019 to 2030 (2). The Thyroid Cancer-Specific Quality of Life Questionnaire (THYCA-QOL) has highlighted concerns with scarring, and a history of minimally invasive thyroid surgery was found to be associated with better thyroid cancer-specific QOL (3). In the pursuit of improved cosmetic outcomes, various endoscopic thyroid surgery approaches have been introduced, with the bilateral areolar approach showing promise and being particularly recommended for lymph node dissection (4–6).

En-bloc resection, originally introduced for radical mastectomy in 1952, provides an alternative technique for resecting solid tumors and lymph-node chains, including those related to head and neck tumors, and thyroid cancers (7–9). However, the safety and feasibility of performing en-bloc resection during thyroidectomy and lymph node dissection are not yet well-established, particularly with the endoscopic method, as there is a lack of long-term follow-up studies (10).

Since January 2010, our institution has implemented the en-bloc method in endoscopic thyroidectomy and lymph node dissection, and the patients have been followed up for an average of ten years after surgery. This observational and retrospective study aims to compare the safety and feasibility of the en-bloc resection method with the conventional method in endoscopic thyroid surgery.

## Materials and methods

### Patients

Between May 2010 and April 2015, 321 patients were retrospectively enrolled at the Department of Thyroid Surgery, Second Affiliated Hospital of Zhejiang University School of Medicine. Among them, 108 patients underwent the modified en-bloc resection (Lobectomy and CND were performed simultaneously), and 213 patients underwent the conventional method (lobectomy was performed first, followed by CND) via bilateral areolar approach (BAA). The inclusion criteria for endoscopic thyroidectomy were: (1) a diagnosis of PTC confirmed by preoperative fine needle aspiration (FNA), (2) dominant tumor size ≤ 2.0 cm, (3) patient ages 15 to 50 years, (4) absence of lateral neck metastasis or distant metastasis based on preoperative evaluation, (5) patients with no evidence of contralateral central and lateral neck metastasis, and (6) pursuit of cosmetic outcome. The exclusion criteria were: (1) body mass index (BMI) > 30, (2) short neck, (3) history of neck or chest surgery, and (4) concurrence of contralateral thyroid cancer. Other inclusion and exclusion criteria for endoscopic thyroidectomy were described in our previous study (11). Informed consent was obtained from all patients, and the study received approval from the Hospital Ethical Committee [Project No. 2019 Annual Review (010)].

### Operative methods

In this study, all patients who were included underwent lobectomy and ipsilateral central node dissection (CND) in endoscopic thyroid surgery via BAA. The boundary of CND was delineated cranially by the superior thyroid arteries, caudally by the innominate vein, laterally by the carotid sheaths, and dorsally by the prevertebral fascia.

**Figure 1:**
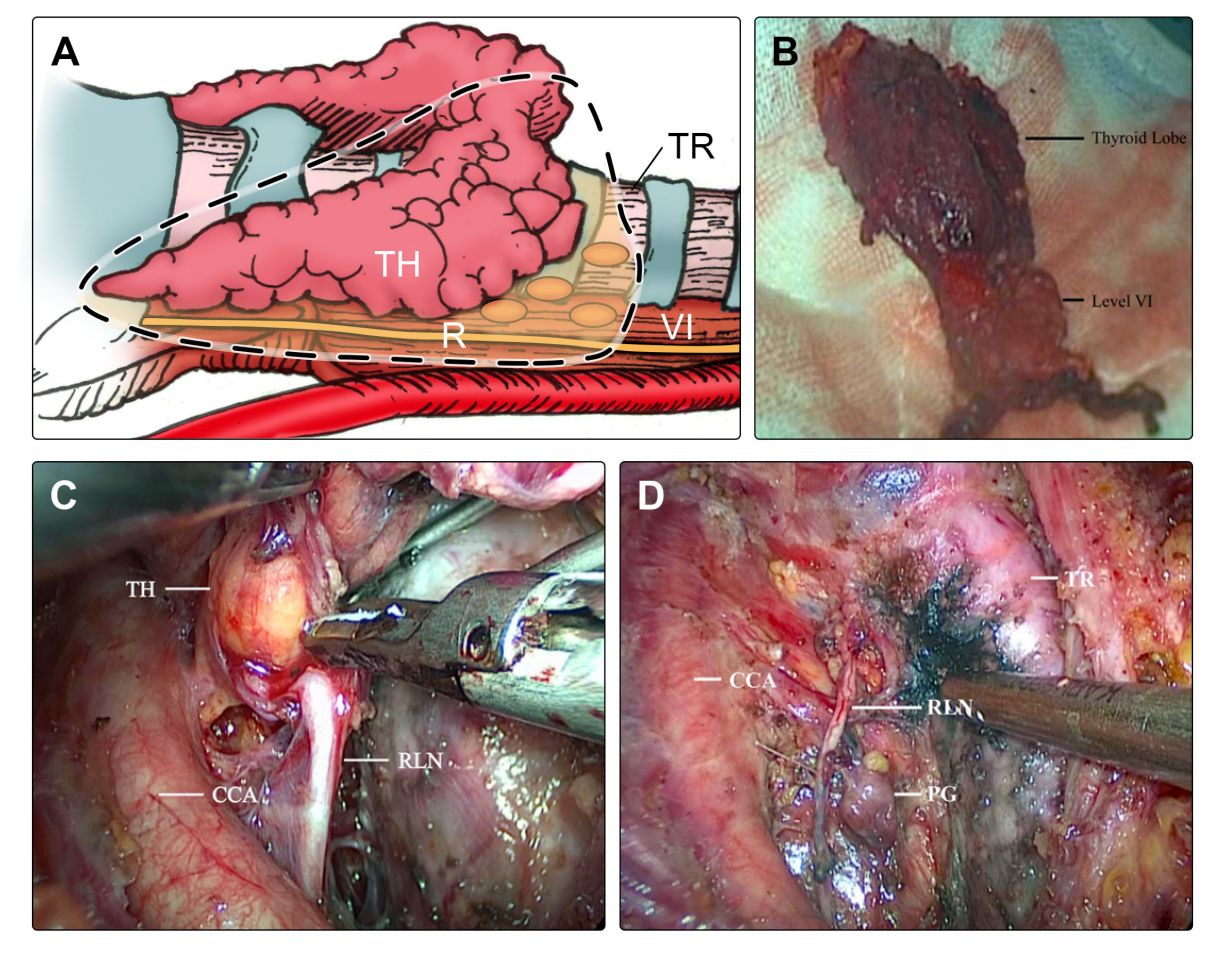
During the surgery of lobectomy and ipsilateral CND in the modified en-bloc group, **(A)** the thyroid lobe and tissues in the central compartment were resected “en-bloc” simultaneously (dotted box). **(B)** The lymph nodes in the central compartment were resected integrally, including pre-tracheal, paratracheal, pre-laryngeal, and post-laryngeal areas. **(C)** Thyroid and lymph node dissections were carefully performed, and **(D)** the RLN and PG were preserved satisfactorily. *TH*, thyroid, *TR*, trachea, *RLN*, recurrent laryngeal nerve, *PG*, parathyroid gland, *CCA*, common carotid artery, *VI*, level VI (central compartment).

In the modified en-bloc group, resection was performed using the “en-bloc” method, which involved the simultaneous removal of the involved structures, including the thyroid lobe and tissues in the central compartment. This was done without any contact with the tumor (**Figure 1**). The resected tissues in the central compartment comprised fibro-fatty tissues, fascia tissues, and lymph nodes including those in the pre-tracheal, para-tracheal, pre-laryngeal, and post-laryngeal areas.

Conversely, in the conventional group, as outlined in a previous study (12), the surgical procedure involved performing thyroidectomy first, followed by the implementation of CND.

### Steps of modified en-bloc resection

The process of modified en-bloc resection via BAA involves several important steps: (1) Pull the strap muscles apart using the upper and lower retractors to create space. (2) Separate the pre-tracheal connective tissue to expose the trachea and identify the attachment of the thymus. Then divide the isthmus along the pre-tracheal fascia to the mid-trachea. (3) Identify and separate the inferior parathyroid gland along the thyrothymic ligament. (4) Separate the lateral border of the thyroid along the front of the common carotid artery to the upper pole. (5) Proceed upward along the cricothyroid space, cut off the anterior branch of the superior thyroid artery, and separate the upper pole. (6) Locate and expose the recurrent laryngeal nerve (RLN) on the inner side of the common carotid artery, resect the central area, and separate the thyroid to the entrance of the RLN using the nerve-tunnel method (6). (7) Complete the en-bloc resection of the thyroid and central compartment and save the superior parathyroid gland by cutting close by the thyroid capsule. (8) Resect the posterior lymph nodes around the recurrent laryngeal nerve when necessary (**Supplementary Video 1**).

### Clinicopathologic characteristics

In the study, various clinicopathologic factors were recorded, including the patient’s gender, age, tumor location, size of the dominant nodule, multifocal lesions, extrathyroidal extension, and the presence of Hashimoto’s thyroiditis. Surgical evaluations included the operative duration, amount of blood loss, number of lymph nodes yield (LNY), and postoperative hospital stay.

The study also assessed vocal cord function using laryngoscope, and measured serum parathyroid hormone (PTH) and calcium levels on the first day and at the six-month follow-up after surgery. These parameters represented transient and permanent vocal cord paralysis, hypoparathyroidism, and hypocalcemia, respectively.

Patients were followed up every three months after surgery for eight to twelve years, with thyroid function and cervical B-mode ultrasound included in each follow-up. Any patients who were found to have recurrence or metastasis during the follow-up period received systematic treatment.

This comprehensive approach allowed for the thorough evaluation of both clinicopathologic factors and surgical outcomes, ensuring a holistic assessment of the patients’ conditions and the effectiveness of the surgical interventions.

### Statistical analysis

The chi-square test was utilized for comparing categorical variables. To analyze continuous variables between the modified en-bloc and conventional groups, the choice between an independent two-sample Student’s t-test or a Wilcoxon test was contingent upon meeting assumptions of normality. Results are presented as mean ± standard deviation (SD) for normally distributed data. Percentages (%) are used for categorical variables. All statistical tests were two-sided, with statistical significance set at P < 0.05. Statistical analyses were conducted using IBM SPSS software, version 22.0 (SPSS, Chicago, IL).

## Results

### Clinicopathologic characteristics

The baseline characteristics of patients in the modified en-bloc group and conventional group are summarized and compared in **Table 1**. Both groups demonstrated similar distributions in gender (P = 0.19), age (P = 0.94), tumor locations (P = 0.91), and the incidence of concomitant Hashimoto’s thyroiditis (P = 0.77). Tumor characteristics also showed no significant difference between the groups in terms of dominant nodule size (P = 0.58), multifocal lesions (P = 0.76), or extrathyroidal extension (P = 0.13). The median follow-up duration for all patients was 10.2 ± 2.1 years.

**Table 1:** Analysis of clinicopathologic characteristics between the modified en-bloc group and conventional group.

Characteristics	Modified en-bloc Group (n=108)	Conventional Group (n=213)	*P* value
Gender (Female %)	105 (97.2%)	200 (93.9%)	0.19
Age (years)	33.7 ± 7.2	33.7 ± 8.0	0.94
Tumor location (Left %)	48 (44.4%)	96 (45.1%)	0.91
Hashimoto thyroiditis, n (%)	17 (15.7%)	31 (14.6%)	0.77
Size of dominant nodule (cm)	0.80 ± 0.58	0.58 ± 0.32	0.58
Multifocal lesions, n (%)	7 (6.5%)	12 (5.6%)	0.76
Extrathyroidal extension, n (%)	21 (19.4%)	28 (13.1%)	0.13

P value represents the difference between the modified en-bloc group and conventional group.

### Analysis of operative outcomes

In this study, 108 patients underwent endoscopic thyroidectomy and ipsilateral CND with modified en-bloc resection, while 213 patients underwent the conventional method without any conversions to open surgery. The surgical outcomes for both groups are presented in **Table 2**.

**Table 2:** Comparison of surgical outcomes in the modified en-bloc group and conventional group.

Surgical outcomes	Modified en-bloc Group (n=108)	Conventional Group (n=213)	*P* value
Operative duration (min)	162.5 ± 38.3	155.0 ± 35.8	0.14
Postoperative hospital stay (d)	4.8 ± 1.5	4.9 ± 1.6	0.58
Blood loss (ml)	11.9 ± 5.5	13.2 ± 7.5	0.13
Lymph node yield (*LNY*)	7.5 ± 4.5	5.6 ± 3.6	< 0.01*
Lymph node metastasis, n (%)	50 (46.3%)	70 (32.9%)	0.02*
Transient *VC* paralysis, n (%)	8 (7.4%)	15 (7.0%)	0.90
Parathyroid transplant, n (%)	24 (22.2%)	41 (19.2%)	0.53
Transient hypocalcemia, n (%)	3 (2.8%)	4 (1.9%)	0.60
Permanent *VC* palsy, n (%)	0 (0%)	0 (0%)	NA
Permanent hypocalcemia, n (%)	0 (0%)	0 (0%)	NA
Metastasis after surgery, n (%)	2 (1.8%)	2 (0.9%)	0.60

*P value represents the difference between the modified en-bloc group and conventional group. VC, Vocal Cord.NA, Not Available.

The mean number of lymph node yield in the central compartment was significantly higher in the modified en-bloc group (7.5 ± 4.5) compared to the conventional group (5.6 ± 3.6) (P < 0.01). Regarding the incidence of parathyroid tissue resected and requiring intramuscular injection (parathyroid transplant), there was no significant difference between the modified en-bloc group (22.2%, 24/108) and the conventional group (19.2%, 41/213) (P = 0.53).

Postoperative pathological findings confirmed a higher rate of lymph node metastases in the central compartment of the modified en-bloc group (46.3%, 50/108) compared to the conventional group (32.9%, 70/213) (P = 0.02). No cases with large metastatic lymph nodes (diameter > 3.0 cm), lymphovascular invasion, or aggressive variants of PTC were observed in either the modified en-bloc group or conventional group. There was no significant difference between the two groups in terms of operative duration (P = 0.14), blood loss (P = 0.13), or postoperative hospital stay (P = 0.58), respectively.

Comparing postoperative complications, including transient vocal cord paralysis (P = 0.90) and hypocalcemia (P = 0.60), revealed no significant differences between the two groups. Notably, no cases of permanent vocal cord paralysis or hypocalcemia were observed during the follow-up period.

In the long-term follow-up (average ten years), tumor recurrence or lateral neck metastasis occurred in two patients each in both the modified en-bloc and conventional groups, showing no significant difference between the groups (1.8% vs. 0.9%, P = 0.60).

### Learning Curve

**Figure 2:**
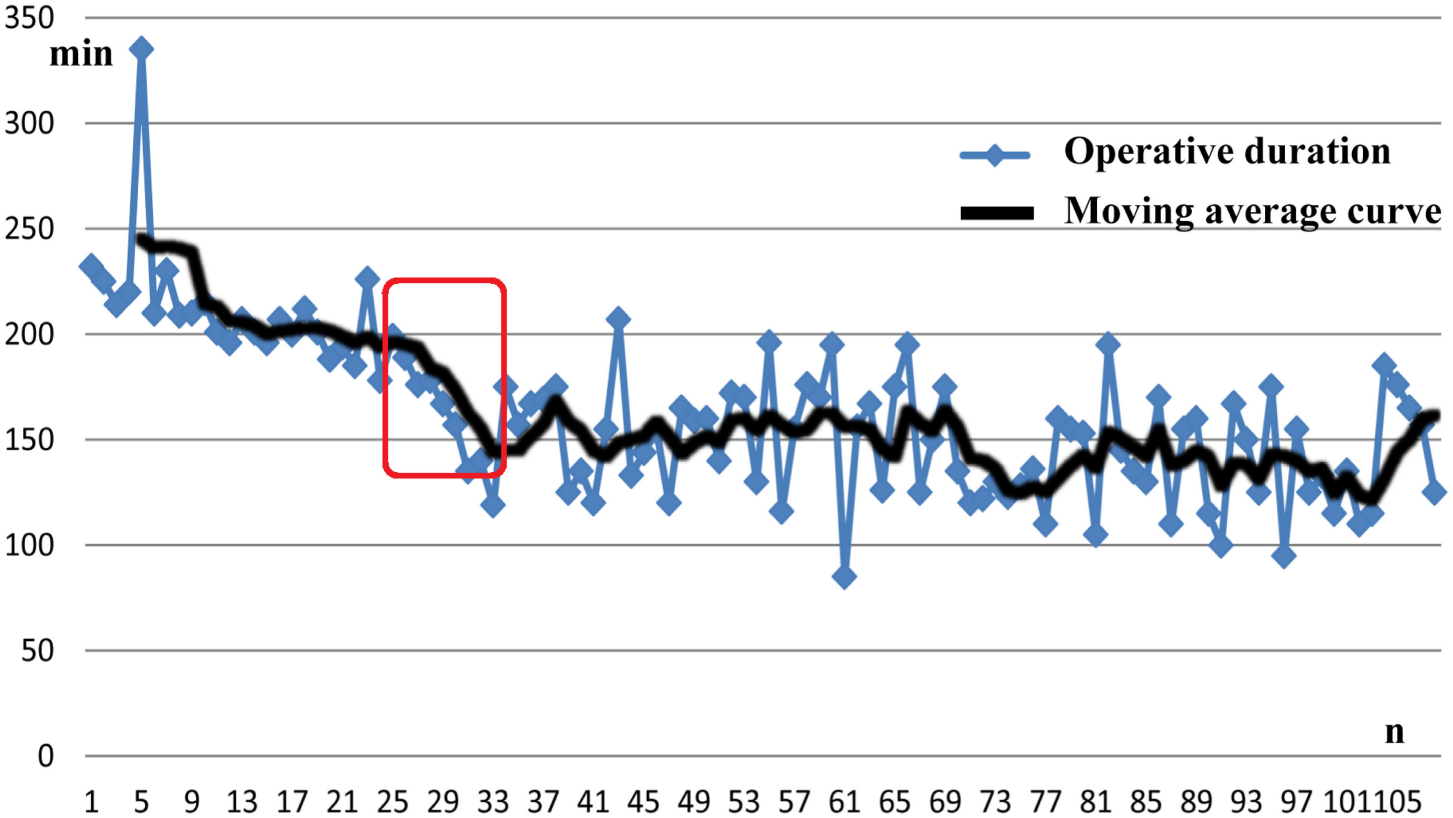
Operative duration, including lobectomy and ipsilateral CND with modified en-bloc method, was markedly decreased after 25-35 cases, which can be considered the learning curve. *CND*, central node dissection.

The learning curve for lobectomy and ipsilateral CND with modified en-bloc resection was assessed in 108 PTC patients between May 2010 and April 2015. These procedures were conducted by a single surgeon who initially had limited experience with the modified en-bloc method in endoscopic thyroidectomy. Evaluation through a moving average curve revealed a notable trend: the operative duration exhibited a significant reduction after the initial 25-35th cases (**Figure 2**).

## Discussion

Advancements in endoscopic technology and surgical instruments have transformed endoscopic thyroidectomy into a safe and viable surgical option for patients diagnosed with PTC (13). Preoperative diagnosis of PTC has been significantly bolstered by advanced examinations such as ultrasound elastography, real-time elastography, and fine-needle aspiration biopsy (14, 15), contributing to more precise diagnostic assessments. The primary objectives of endoscopic thyroidectomy techniques include achieving optimal efficacy and safety while concurrently improving perioperative outcomes and cosmetic results (16). Previous studies, including our own, have showcased the feasibility of this technique, particularly in treating papillary thyroid non-microcarcinoma (PTNMC), leveraging intraoperative neuromonitoring and energy-based devices (17–19). However, the majority of earlier trials, including our own prior investigations, were constrained by shorter-term follow-up periods in assessing disease-free status (20–22). The present study delves into a comprehensive, long-term institutional post-surgical follow-up, spanning an average of ten years. This extended follow-up period aims to provide a more accurate estimation of recurrence or metastasis rates, offering deeper insights into the efficacy and durability of the surgical outcomes.

Long-term follow-up is essential for assessing relapse-free survival (RFS) and disease-free survival (DFS) (23, 24). Over the ten-year follow-up period, our study observed recurrence/metastasis in four patients. One patient from each group (modified en-bloc and conventional) developed new lesions in the contralateral thyroid lobe, confirmed as PTC by FNA, leading to a second open surgery of total thyroidectomy and bilateral central node dissection. Additionally, one patient from each group was identified with lymph node metastasis in the lateral cervical region through cervical B-mode ultrasound. Both underwent a second open surgery involving total thyroidectomy, bilateral central node dissection, and unilateral lateral neck dissection. Notably, no residual lesion was detected in the thyroid or central neck area during the long-term follow-up, indicating the efficacy of endoscopic surgery for thyroidectomy and central neck dissection. Additionally, our findings revealed no statistically significant difference in recurrence/metastasis between the modified en-bloc and conventional group, suggesting that en-bloc represents a viable alternative method in endoscopic thyroidectomy.

En-bloc resection is an appropriate approach aligned with the oncologic concept (8) and has been practiced in thyroid surgery at our institution since January 2010 (25). Our current study revealed a notable increase in the number of lymph nodes obtained in the modified en-bloc group (7.5 ± 4.5) compared to the conventional group (5.6 ± 3.6) (P < 0.01). Furthermore, the incidence of lymph node metastasis was significantly higher in the modified en-bloc group (50/108, 46.3%) than in the conventional group (70/213, 32.9%) (P = 0.02). Notably, there was no significant difference in the recurrence/metastasis rate comparison between the modified en-bloc group and the conventional group. These findings affirm that en-bloc resection is an effective method for central neck dissection during endoscopic thyroidectomy.

Preserving the inferior parathyroid gland can present a challenge, particularly when it is situated amidst paratracheal lymph nodes during endoscopic thyroidectomy, leading to a contentious issue (26). In this study, we introduced the modified en-bloc dissection method in endoscopic thyroid surgery to assess whether it heightens the difficulty of preserving the parathyroid gland and its blood vessels. Our results indicated that the incidence of parathyroid tissue resected and necessitating intramuscular injection (parathyroid transplant) was comparable in both the modified en-bloc group (22.2%, 24/108) and the conventional group (19.2%, 41/213) (P = 0.53). Furthermore, there was no significant difference in the rate of transient hypocalcemia between the modified en-bloc group (3/108, 2.8%) and the conventional group (4/213, 1.9%). Therefore, our findings suggest that en-bloc resection in endoscopic thyroid surgery does not increase the incidence of parathyroid gland injury.

Our comparative analysis investigating modified en-bloc resection versus the conventional method in endoscopic thyroid surgery yielded no statistically significant disparities in postoperative complications. Notably, rates of transient or permanent vocal cord paralysis and hypocalcemia demonstrated similarity between both groups. Within our study cohort, neither surgical method led to complications such as recurrence around the subcutaneous tunnel, subcutaneous emphysema, tracheal injury, or Horner’s syndrome. Additionally, all endoscopic surgeries within both the modified en-bloc and conventional groups were executed satisfactorily, without necessitating any conversions to open surgery. The findings suggest that modified en-bloc resection bears comparable rates of surgical complications to the conventional method in endoscopic thyroid surgery. Consequently, based on these results, modified en-bloc resection emerges as a potentially safe alternative, demonstrating akin outcomes to the conventional method.

The adoption of modified en-bloc resection via BAA in endoscopic thyroid surgery necessitates a learning curve due to its novelty as an operating method. This curve is characterized by criteria including A) operative time, B) blood loss, and C) the number of harvested lymph nodes (27). In our study, a surgeon with limited prior experience in modified en-bloc resection completed the learning curve after conducting 35 operations, demonstrating a notable reduction in surgical time coupled with enhanced consistency. These outcomes are in alignment with observations from other surgical centers. Studies by Dabsha, et al. highlighted the statistical significance in development of the learning curve for endoscopic thyroidectomy via trans-oral and trans-axillary approaches, with the learning curve ranging between 6 and 15 annual cases (28). Kandil, et al. reported that compared to trans-axillary endoscopic thyroidectomy, BAA appeared to entail a less steep learning curve, requiring approximately 69 cases for proficiency (29). Moreover, technical indices such as operative duration, blood loss, and postoperative hospital stay exhibited similarity between the modified en-bloc and conventional groups in our study. This finding underscores the feasibility of employing en-bloc as a viable procedure in endoscopic thyroidectomy.

**Figure 3:**
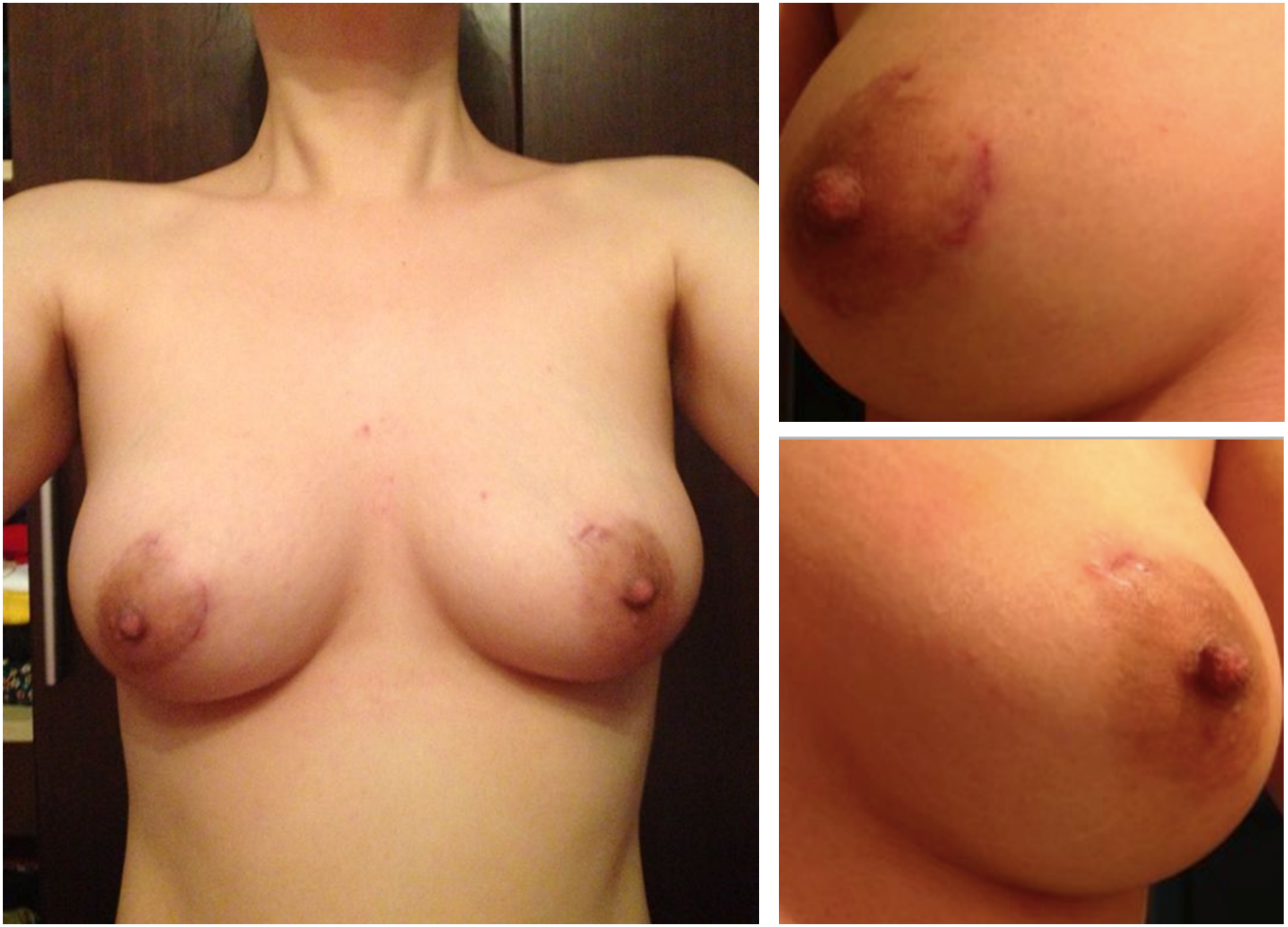
Macroscopic view of breast one month after endoscopic thyroid surgery via bilateral areola approach showing practically no scars.

The preservation of Quality of Life (QOL) post-thyroid surgeries is a paramount concern (30). The presence of neck scars can impose substantial psychological burdens on patients, and prior research in minimally invasive thyroid surgery has associated it with enhanced thyroid cancer-specific QOL (3). Notably, the cosmetic outcome of BAA, characterized by the absence of visible neck scars, demonstrated excellent results. Moreover, scars in the mammary areolas were nearly imperceptible one month postoperatively (**Figure 3**). Furthermore, preliminary investigations have indicated that patients undergoing BAA exhibited lower levels of scar self-consciousness, higher levels of cosmetic satisfaction, and an improved QOL compared to those undergoing open surgeries (data not included in this study). The absence of visible scars, particularly on the neck, represents a significant advantage of BAA, potentially addressing the psychological burdens associated with scarring following thyroid surgeries. Although further research is warranted to validate these preliminary observations, the initial indicators underscore the potential of endoscopic thyroid surgery via BAA in positively impacting patients’ postoperative QOL.

The present study has some limitations that warrant consideration when interpreting the findings. Firstly, its retrospective nature, confined to a single institution, may restrict the extrapolation of results to broader settings. Secondly, the resection parameters for thyroidectomy and prophylactic CND were aligned with the Chinese national guidelines for treating thyroid cancer (31), potentially offering more applicability to the Asian population. To address these constraints and facilitate a more comprehensive analysis, future investigations would benefit from well-designed, prospective, multicenter, and randomized controlled clinical trials. Such studies would offer greater generalizability and elucidate the broader applicability of the modified en-bloc method and the BAA approach. However, despite these limitations, our study stands out for its substantial sample size of patients undergoing endoscopic thyroid surgery via BAA. Additionally, the extended long-term follow-up period (average 10 years) underscores the commitment to evaluate the effectiveness of the modified en-bloc method and the BAA approach in clinical practice.

In summary, our study affirms the technical feasibility, safety, and effectiveness of employing modified en-bloc resection in endoscopic thyroid surgery via BAA approach. The procedure yields excellent cosmetic outcomes, positioning it as a viable surgical option for specific patients diagnosed preoperatively with a dominant diameter of ≤ 2cm, devoid of metastasis, and expressing concerns regarding cosmesis. However, to solidify these observations and ascertain the long-term efficacy and safety of this approach, further prospective, multicenter, and randomized controlled trials are imperative. These trials will substantiate the validity of our findings and facilitate the establishment of modified en-bloc resection via BAA as a standardized and validated approach in the management of select PTC cases.

## Data availability statement

The raw data supporting the conclusions of this article will be made available by the authors, without undue reservation.

## Ethics statement

The studies involving humans were approved by the 2nd Affiliated Hospital of Zhejiang University, School of Medicine. The studies were conducted in accordance with the local legislation and institutional requirements. The participants provided their written informed consent to participate in this study. Written informed consent was obtained from the individual(s) for the publication of any potentially identifiable images or data included in this article.

## Author contributions

QX: Writing – original draft. JM: Data curation, Writing – original draft. YD: Data curation, Writing – original draft. LL: Formal Analysis, Writing – original draft. RZ: Investigation, Writing – original draft. DL: Software, Writing – original draft. PW: Writing – review & editing. XY: Writing – original draft, Writing – review & editing.

## Supplementary material

Supplementary Video 1 Surgical steps of modified en-bloc resection with endoscopic thyroidectomy via BAA (lobectomy and ipsilateral central node dissection). *BAA*, bilateral areolar approach.

## References

[1] Megwalu UC Moon PK. Thyroid cancer incidence and mortality trends in the United States: 2000-2018. Thyroid. (2022) 32:560–70. doi: 10.1089/thy.2021.0662 35132899

[2] Wu J Zhao X Sun J Cheng C Yin C Bai R. The epidemic of thyroid cancer in China: current trends and future prediction. Front Oncol. (2022) 12:932729. doi: 10.3389/fonc.2022.932729 36119514 PMC9478365

[3] Chen C Cao J Wang Y Han X Zhang Y Zhuang S. Health-related quality of life and thyroid cancer-specific symptoms in patients treated for differentiated thyroid cancer: a single-center cross-sectional survey from mainland China. Thyroid. (2023) 33:474–83. doi: 10.1089/thy.2022.0490 36792949

[4] Sun P Zhan J Chong TH Li J Wang C. Endoscopic central lymph node dissection of thyroid cancer *via* chest-breast approach: results, indications, and strategies. Surg Endosc. (2022) 36:4239–47. doi: 10.1007/s00464-021-08758-7 35169879

[5] Yu X Jiang Y Li Y He Q Pan L Zhu P. Comparison of different mandibular jawlines classifications on transoral endoscopic thyroidectomy for papillary thyroid carcinoma: experiences of 690 cases. Front Endocrinol (Lausanne). (2022) 13:842148. doi: 10.3389/fendo.2022.842148 35250890 PMC8891504

[6] Yu X Li Y Liu C Jiang Y Liu Z He Q. Effects of intraoperative neural tunnel in protecting recurrent laryngeal nerve: experiences in open, trans breast, and transoral endoscopic thyroidectomy. Front Oncol. (2022) 12:779621. doi: 10.3389/fonc.2022.779621 35280753 PMC8904970

[7] Urban JA Baker HW. Radical mastectomy in continuity with en-bloc resection of the internal mammary lymph-node chain; A new procedure for primary operable cancer of the breast. Cancer. (1952) 5:992–1008. doi: 10.1002/1097-0142(195209)5:5<992::aid-cncr2820050515>3.0.co;2-z 12988188

[8] Sheng X Liu J Fang J Zheng X Wang S. En-bloc resection of total thyroid and bilateral central compartment lymph nodes *via* a gasless transoral approach in papillary thyroid carcinoma. Front Endocrinol (Lausanne). (2023) 14:1130791. doi: 10.3389/fendo.2023.1130791 36923227 PMC10009257

[9] Zanoletti E Marioni G Nicolai P Mazzoni A. The contribution of oncological lateral skull base surgery to the management of advanced head-neck tumors. Acta Otolaryngol. (2023) 143:101–5. doi: 10.1080/00016489.2023.2174270 36883304

[10] Barranco H Fazendin J Lindeman B Chen H Ramonell KM. Thyroid hormone replacement following lobectomy: long-term institutional analysis 15 years after surgery. Surgery. (2023) 173:189–92. doi: 10.1016/j.surg.2022.05.044 36202649

[11] Yan HC Xiang C Wang Y Wang P. Scarless endoscopic thyroidectomy (set) lateral neck dissection for papillary thyroid carcinoma through breast approach: 10 years of experience. Surg Endosc. (2021) 35:3540–6. doi: 10.1007/s00464-020-07814-y 32691204

[12] Yu X Liu C Yan M Gong W Wang Y. Hyperthermal liquid, spray, and smog may be potential risk factors for recurrent laryngeal nerve thermal injury during thyroid surgeries. Endocrine. (2021) 72:198–207. doi: 10.1007/s12020-020-02451-w 32779090

[13] Dong F Yang A Ouyang D. Retroauricular single-site endoscopic thyroidectomy-a balanced endoscopic approach for thyroid excision. JAMA Surg. (2023) 158:548–9. doi: 10.1001/jamasurg.2022.7723 36753130

[14] Sengul I Sengul D. Hermeneutics for evaluation of the diagnostic value of ultrasound elastography in TIRADS 4 categories of thyroid nodules. Am J Med Case Rep. (2021) 9:538–9. doi: 10.12691/ajmcr-9-11-5

[15] Sengul D Sengul I. Reassessing combining real-time elastography with fine-needle aspiration biopsy to identify Malignant thyroid nodules. Am J Med Case Rep. (2021) 9:552–3. doi: 10.12691/ajmcr-9-11-9 PMC7747118 33327813

[16] Kim DH Kim SW Kim GJ Basurrah MA Hwang SH. Efficacy and safety of minimally invasive thyroid surgery: a network meta-analysis. Laryngoscope. (2023) 133(10):2470–9. doi: 10.1002/lary.30645 36892037

[17] Liu Z Li Y Wang Y Xiang C Yu X Zhang M. Comparison of the transoral endoscopic thyroidectomy vestibular approach and open thyroidectomy: a propensity score-matched analysis of surgical outcomes and safety in the treatment of papillary thyroid carcinoma. Surgery. (2021) 170:1680–6. doi: 10.1016/j.surg.2021.06.032 34284897

[18] Li Y Liu Z Song Z Wang Y Yu X Wang P. Comparison of the endoscopic thyroidectomy *via* areola approach and open thyroidectomy: a propensity score matched cohort study of 302 patients in the treatment of papillary thyroid non-microcarcinoma. Front Oncol. (2023) 13:1081835. doi: 10.3389/fonc.2023.1081835 36925920 PMC10012860

[19] Sengul D Sengul I Ozturk T. Sutureless thyroidectomy with intraoperative neuromonitoring and energy-based device without sternotomy for symptomatic substernal goiter harboring thyroiditis of gland parenchyma. Cureus. (2021) 13. doi: 10.7759/cureus.16258 PMC8269993 34277302

[20] Nguyen KA Nguyen ND. Transoral endoscopic thyroidectomy with or without central neck dissection. Am J Otolaryngol. (2023) 44:103728. doi: 10.1016/j.amjoto.2022.103728 36495646

[21] Li Y Liu Z Wang Y Yu X Wang T Xiang C. Is transoral endoscopic thyroidectomy safe for total thyroidectomy compared to open thyroidectomy? A propensity-score matched cohort study with papillary thyroid carcinoma. J Surg Oncol. (2023) 128(4):502–9. doi: 10.1002/jso.27360 37303249

[22] Xie QP Xiang C Wang Y Yan HC Zhao QZ Yu X. The patterns and treatment of postoperative hemorrhage and hematoma in total endoscopic thyroidectomy *via* breast approach: experience of 1932 cases. Endocrine. (2019) 63:422–9. doi: 10.1007/s12020-018-01837-1 30652236

[23] Watanabe K Igarashi T Uchiyama M Ojiri H. Relapse-free survival after adjuvant radioactive iodine therapy in patients with differentiated thyroid carcinoma with a microscopically positive tumor margin. Ann Nucl Med. (2020) 34:920–5. doi: 10.1007/s12149-020-01523-1 32940889

[24] Kawamoto T Shikama N Fukumori T Hoshi M Yamada T. Long-term clinical outcomes and prognostic factors for patients with papillary thyroid carcinoma with other organ invasions after adjuvant radioactive iodine. Endocrine. (2023) 80:79–85. doi: 10.1007/s12020-022-03251-0 36367673

[25] Li Z Wang P Wang Y Xu S Cao L Que R. Endoscopic lateral neck dissection *via* breast approach for papillary thyroid carcinoma: a preliminary report. Surg Endosc. (2011) 25:890–6. doi: 10.1007/s00464-010-1292-7 20734078

[26] Xu W Teng C Ding G Zhao N. Hypoparathyroidism risk after total endoscopic thyroidectomy for papillary thyroid cancer: a comparison of the transoral vestibular and breast approaches. Cancer Manag Res. (2022) 14:2485–92. doi: 10.2147/CMAR.S380024 PMC9391930 35996659

[27] Kim KH Ji YB Song CM Kim E Kim KN Tae K. Learning curve of transoral robotic thyroidectomy. Surg Endosc. (2023) 37:535–43. doi: 10.1007/s00464-022-09549-4 36002679

[28] Dabsha A Khairallah S Elkharbotly I Hossam E Hanafy A Kamel M. Learning curve and volume outcome relationship of endoscopic trans-oral versus trans-axillary thyroidectomy; A systematic review and meta-analysis. Int J Surg. (2022) 104:106739. doi: 10.1016/j.ijsu.2022.106739 35764254

[29] Kandil E Akkera M Shalaby H Munshi R Attia A Elnahla A. A single surgeon's 10-year experience in remote-access thyroid and parathyroid surgery. Am Surg. (2021) 87:638–44. doi: 10.1177/0003134820950300 33142070

[30] Watt T Christoffersen T Brogaard MB Bjorner JB Bentzen J Hahn CH. Quality of life in thyroid cancer. Best Pract Res Clin Endocrinol Metab. (2023) 37:101732. doi: 10.1016/j.beem.2023.101732 36732089

[31] C.O.T.P. Health. National guidelines for diagnosis and treatment of thyroid cancer 2022 in China (english version). Chin J Cancer Res. (2022) 34:131–50. doi: 10.21147/j.issn.1000-9604.2022.03.01 PMC9273579 35873884

